# Swann's Way: Youth, Personal Affinities, and Acculturation Through Sport in Nineteenth Century France

**DOI:** 10.3389/fspor.2020.561542

**Published:** 2021-02-04

**Authors:** François Bourmaud

**Affiliations:** ^1^Centre d'Histoire du XIX° Siècle (CRHXIX), Sorbonne Université, Paris, France; ^2^Centre d'Etudes Olympiques et de la Globalisation du Sport (CEO&GS), UNIL, Lausanne, Switzerland

**Keywords:** sports diffusion, cultural mediators, France, youth, sport history, Swann, Satgé

## Abstract

This study of two Franco-British cultural mediators and their entourage explores the criteria of age and personal affinities in the process of acculturation through sports in nineteenth century France. The youth of those who were the first to take up on modern sports is an element that at first seems obvious, but it is probable that up until now this has been underestimated in the understanding of the way individuals opened up to embrace these new activities. Nevertheless, this factor is powerful, in particular when crossed with personal links such as friendship or camaraderie which prompt the sharing and discovery of sporting activities. Despite their dual culture, Alfred Swann (1863–1928) and Cosme de Satgé (1840–1898) did not adopt British pastimes with the same enthusiasm: the former discovered modern sport when he was an adolescent and became an active agent in their spread in Paris whereas the latter did not manage to truly appreciate and pass on British Leisure activities he discovered after he had turned 30. Moreover, both cases underline the fact that sports diffusion often follows the channels of personal affinities: the young lycée pupils around Alfred Swann, like Cosme de Satgé's children, were acculturated through British sports at the time of their adolescence, not by adults, but by friends and chums of their age.

## Introduction

Studies on the spread of sport throughout the world are currently the focus of a certain interest. Some of them are focused on one sport in particular while others feature geographical areas (Eisenberg, 1999; Van Bottenburg, 2001; Darbon, 2008; Dietschy, 2011; Holt, 2011; Tomlinson and Young, 2011, Van Bottenburg, 2010). They attempt to analyse both the conditions which make these cultural transfers possible and the actors who take part but also they examine the changes that ensue. The aim of this article is to contribute to a better understanding of this dissemination mechanism by underlining the point that in studies on sports diffusion the criterion of age needs to be taken into account as well as the relationships between the individuals concerned.

The youth of those who were the first to take up modern sports is in fact an element that at first sight seems obvious but it is probable that up until now this has been underestimated in the understanding of the way individuals opened up to embrace these new activities. Nevertheless, this factor is powerful, in particular when crossed with personal links such as friendship or camaraderie which prompts the sharing and discovery of sporting activities.

In France the introduction of modern British sports in the second half of the nineteenth century is an interesting case for the illustration of the importance of youthfulness and personal affinities when it comes to the spread of sport. First, we have chosen to focus on a pioneering group, the Société des Courses du Bois de Boulogne (The Bois de Boulogne Running Club) which was one of the first in Paris where athletic sports as they were called were played, in particular running and football. Around the central figure of Alfred Thomas Swann (1863–1928), who inspired Marcel Proust for the famous first volume of À* la recherche du temps perdu*^1^. *Remembrance of Things Past*, translation by C. K. Scott Moncrieff and Terence Kilmartin, London, Chatto and Windus, 1922 *(In Search of Lost Time)*, pupils from the prestigious Lycée Condorcet school were introduced to these physical activities from across the English Channel between 1879 and 1880. Through a sociological and a cultural approach, we propose to study this group of young chums, thus more closely examining the question about “first contact” (Darbon, 2008) which begins the process of the dissemination of sports. In the second part we will compare this study with that of an older person, Cosme de Satgé (1840–1898), whose discovery of physical sports from across the Channel can be examined through his personal diary^2^. This counterpoint and the relative failures that he shows in terms of acculturation therefore bring the focus back to the criterion of age and the importance of camaraderie and friendship in the study of the spread of sports.

Our two case studies rely on different sources. Cosme de Satgé's personal diary is a 4,000 pages first-hand account of his life from 1873 to 1897. It is now part of the family archives kept by his great grandson, Jeremy de Satgé, in London. As for Alfred Swann, we could not find any primary sources apart from his portrait also located in London and retained by his descendence. It is however possible to reconstruct part of his adolescent and adult life through civil status documents, as well as school and clubs archives. Moreover, local and national newspapers were particularly useful to cross-reference our primary and secondary sources: they both provide valuable information to complete and enlarge Cosme de Satge's assertions included in his journal on the one hand and bring testimonies from Alfred Swann's schoolmates on the other hand.

## The Organization of the Société des Courses du Bois de Boulogne (Bois De Boulogne Running Club) Around Alfred Thomas Swann

### A Pioneering Club in 1879

The history of the introduction of athletic sports in Paris is largely bound up with that of the Bois de Boulogne and in particular with that of its oldest club, the Racing Club de France. It was in fact in this vast open space laid out during the Second Empire, that lycée pupils founded a sports society in 1882, using the paths and lawns in the Bois before fixing on a spot called the Croix-Catelan in 1886. If the Racing Club de France has claimed since the end of the nineteenth century that it is the oldest for the practice of athletic sports in Paris it is above all because it managed to establish itself and has lasted over time. However, the first histories of sports played in the capital show that before the Racing Club's foundation a large number of young people's groups, more or less informal, used to meet up on the same grass bordering the Cercle des Patineurs (Skaters' Circle) at the end of the 1870's^3^: the Club des Coureurs (Runners' Club) (1875–76), the Cercle de Madrid (1876), the Paris Football Club (from 1877, with only British members), the Foot-Ball Club of Ecole Monge (1878), or even the Société Sans Nom (Club with No Name) (1880) were all relatively fleeting pioneering groups at the time the Racing Club started in 1882.

Among these unknown groups the Société des Courses du Bois de Boulogne (Bois de Boulogne Running Club) is one of the few cases where witness accounts still survive. In the autumn of 1902 when the Paris daily paper *La Presse* published a series of sports articles on the beginnings of the Racing Club de France authored by Gustave Lafreté, a journalist, one Georges Bertrand wrote to the paper to inform him of the existence of an “older club.” This letter, reproduced in its entirety in the 18 November edition is a remarkable source for it precisely details the manner in which a certain number of boys from the Lycée Condorcet would meet up in the Bois de Boulogne to have running races or to play football.

After having drawn up a list of the members of this society, Georges Bertrand, came to the key role played by a certain person in the acculturation process of British sports: “nearly all the merit, in my opinion, is due to Alfred Swann, who, upon his return from his annual holiday in Brighton knew how to inculcate in us the first rules concerning methodological and reasoned training which he had seen in action in England and in 1879–1880 he formed us into football teams; he transmitted to us his sacred flame” (La Presse, november 18, 1802).” From our point of view this passage is of particular interest for it clearly identifies him as what historians call a cultural mediator.

### Alfred Thomas Swann: The Duality of a Young Man

The history of cultural transfers has long taken an interest in those persons who play the role of mediators, who are known in the English-speaking world as “cultural go-betweens” or “brokers” (Cooper-Richet, 2013). These people are intermediaries who capture a cultural product in a geographical area or a social space and then transmit it in full awareness or unwittingly to others. In the case of the spread of sports, a few remarkable personalities have already been the subject of detailed studies, such as the British missionary and educator Cecil Tyndale-Biscoe who introduced many Indian pupils to modern sports in the Kashmir region (Mangan, 2003). The sporting acculturation of non-British peoples in the nineteenth century is not immediately clear. In order to be passed on to new players, the sporting activities codified in Britain must be mastered by those individuals who wish moreover to share them with neophytes. However, knowing a sport and the desire to pass it on are not self-evident: while the first wish was shared by a growing number of Britons who traveled abroad in the nineteenth century, the second case was rather more exceptional in many countries. This reveals the importance of the cultural mediators.

**Figure 1:**
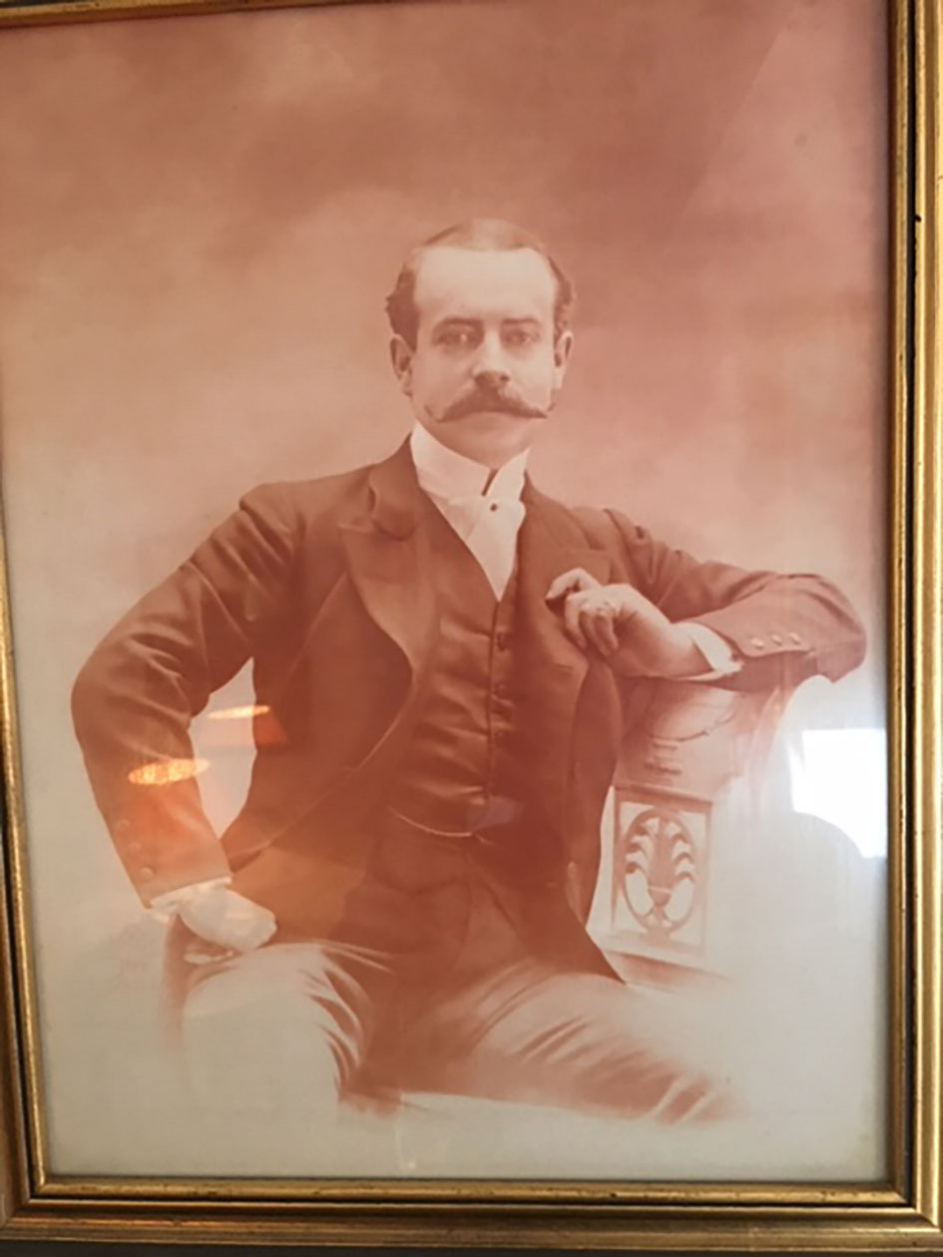
Portrait of Alfred Swann, photo courtesy of Yvonne Swann.

In the case of France, as with many countries in the former British Empire (formal and informal), a certain number of characters have been identified but few of them have been the subject of in-depth studies or works centered on their role as cultural mediators. Moreover, while these studies have been on Georges de Saint-Clair (Bourmaud, 2020), Paschal Grousset (Lebecq, 2013) or even Pierre de Coubertin (Clastres, 2010) where Paris was concerned, they focused mainly on vertical transmissions, which is to say from adults down to young players. The case of the Société des Courses du Bois de Boulogne, where one of the lycée pupils played the role of mediator for his classmates therefore encourages us to take an interest in the person of Alfred Thomas Swann (1863–1928) (Figure 1).

He really was an Anglo-Parisian in that Alfred was a young man with a dual cultural identity, both French and British. He was the son of an English pharmacist Henry Hind Swann (1823–98) and a Parisian mother Judith Félicie Davoust (1830–1903). His father was born in Grantham, in the United Kingdom and he studied medicine in London before settling in Paris in 1847. He opened a renowned pharmacy in Rue Castiglione and quickly became well-known for the sale of medicines sold in the then novel form of pills. Moreover, Swann's father was particularly famous for his skill in the use of chloroform as an anesthetic, the techniques of which had recently been perfected on the other side of the Channel. When the Empress gave birth in 1856 his intervention in alleviating her pain linked to a fracture of the pelvis ensured his brilliant social and professional integration which had been hinted at 3 years previously when he had married the granddaughter of a Marshal of the French Empire (Diehl, 2016). In 1863 an only son, Alfred Thomas Swann^4^ was born to the couple.

The story of this person reflects his ancestry, both British and French, with both cultures constituting two sides of the same coin. Swann was most definitely French since he lived his life south of the Channel. Born in Paris on the 15th of April 1863, he grew up in the very select 16th arrondissement^5^, between the Bois de Boulogne and the Lycée Condorcet which he attended at the end of the 1870's and in the early 1880's. Alfred remained a lifelong Parisian all and spent his life between the capital and the Calvados area where the family manor of Cantepie was located (in Cambremer), as well as spending time in holiday destinations between Houlgate and Cabourg (Le Gaulois, June 11, 1899). It must be said that Swann lived a life of great luxury. On his death his father left him a fortune of around 40,000 pounds, which was about a million francs^6^, which allowed him to live off the income of his investments and to frequent artistic circles in Paris. Alfred was a member of many literary societies in the capital^7^, and his friendships with the journalist Georges Bourdon, the writer Jules Renard [who dedicated his work “Le Rêve” (The Dream) to him in 1906] (Renard, 1906), also the painter Henri Morisset and the sculptor Raymond Bigot, made him part of Parisian high society. He was also one of the contributors to the very select review Le Guide du Concert (The Concert Guide) in which he introduced new composers, in particular Russians^8^. His marriage in 1885 to the charming young Parisian, Suzanne Guillaume, bears testimony to his attachment to France^9^.

However, English blood flowed through Swann's veins. Apart from his surname, which his friend Proust had noted, he spoke the language of Shakespeare perfectly, thanks to his father who often accompanied him on his holiday trips to seaside resorts in the south of England as a young man. There was thus an emotional attachment that crossed the Channel, as is borne out by his desire in June 1894 to keep his father's nationality and to give up French nationality which the law of 22 July 1893 would otherwise have allowed him to have, because of the jus soli (born in the territory) as well as the jus sanguini (blood right) through his mother^10^. His only son, born in 1898 had an English first name, Cecil, and remained a British subject. Alfred Thomas Swann was thus the incarnation of an Anglo-Parisian type, a truly “dual young man” according to the expression used by Christophe Charle to describe these intermediate people who “belong to two cultural levels that are normally separate and who act as go-betweens^“^ (Charle, 1992, p. 75).

### Alfred Swann: A Cultural Go-Between

This dual culture bestowed on him the role of sports mediator. The holidays spent on the Sussex beaches, at Brighton in the 1870's meant that he learned about running races and also football as is evidenced when, upon his return, he took up a coaching role at the Société des Courses du Bois de Boulogne. However, although from 1882 onwards Swann also took part in setting up the Racing Club, he did not practice these two sports, preferring to be an honorary member although he was only 20 years old in 1883. His interest at the time was in lawn tennis. Alongside Georges de Saint-Clair, he played an active part in introducing this game to the shady lawns of the Croix-Catelan in 1886 (Bourdon, 1906). He became a member of the lawn tennis committee of the club that same year and remained in this position until 1889, having been the president between 1890 aand 1894^11^. Lastly, he was also one of the founders of the Racing Club in Cabourg, which from 1893 onwards organized competitions on the Normandy beaches during the summer season (Le Sport, August 9, 1893). It is quite possible that Alfred Swann discovered this racket sport on British beaches and we can see quite clearly how the fact of belonging to two different cultures allowed him to be a sports go-between. He was definitely not a top player nor a great leader but he was an essential component that began the transfer mechanism by establishing a bridge between the two shores of the Channel. While others were responsible for spreading the sport, he was at the origin, transmitting the “sacred flame.” Cultural transfer is not however dependent on just one man; there is an exchange and interaction between two parties and this is why the group of lycée pupils around Alfred Swann in 1879–1880 needs to be considered more closely.

## A Cultural Transfer Between Friends at Lycée Condorcet

Alongside Alfred Swann, the Société des Courses du Bois de Boulogne included a group of young lycée students. In his 1902 letter Georges Bertrand gives a few names: “If I looked, I would certainly find a few of the programmes of these meetings which include the names of a few members who went on to become members of the ‘Racing,’ in particular the Carvallo brothers and Alfred Swann. To these names I could add all those with whom I have kept in touch since that sadly far-off time! Forget, Riollet, Vallarché. The Grant brothers, gone to America, Gaston, Lavoisier, Lacroix are all former sporting chums” (La Presse, november 18, 1802). This list of young men who were eager to discover British sports from Swann poses the question of the responsiveness to a cultural product, in other words the desire to embrace it or not.

In this case, there does not seem to have been any notable resistance to the acculturation by British physical practices, on the contrary. Thus, the particular environment in which the youth of the Société des Courses du Bois de Boulogne lived needs to be examined as well as their age and the affinities between them in order to understand the implementation of the early and easy spread of these sports.

### The Originality of the Lycée Condorcet in the Paris School System

**Figure 2:**
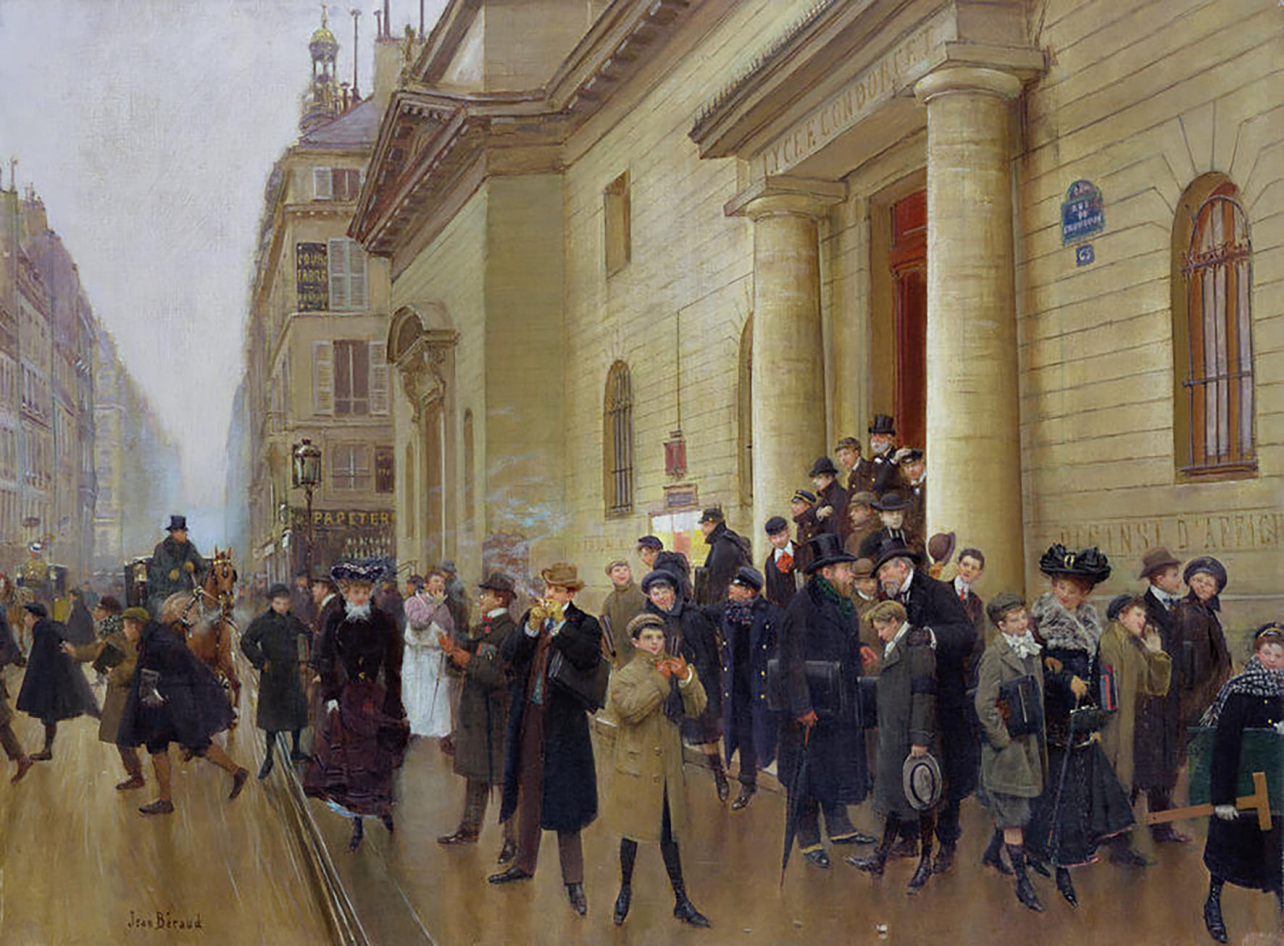
La sortie du lycée Condorcet, jean Béraud, 1903, Musée Carnavalet, Paris.

The first point that these young men had in common was that they all attended the Lycée Condorcet. Among the educational establishments that catered to the elite of the capital this school had a certain originality (Albertini, 2017) (Figure 2).

In the first instance it is a particularly prosperous and elitist establishment. With 1,600 pupils in 1880 (the largest in France; Dupont-Ferrier, 1913, p. 140), Condorcet was the only lycée on the Right Bank, along with the Lycée Charlemagne, and its situation in the heart of the smartest neighborhood, in the 9th arrondissement, meant that its catchment area was very posh^12^. Fees were the highest in Paris, rising to 1,000 francs a year, for each school level. This was about the annual income of a skilled workman or a primary school teacher. In his social study of school pupils in 1867, Pierre Albertini demonstrates how the aristocracy and the upper middle class made up the principal recruitment base for the institution (Albertini, 2017, p. 22). Some professions dominate among the pupils' parents, in particular doctors, lawyers and bankers. In this socio-economic environment the young sportsmen were no exceptions to this rule: for example, Swann was the son of a pharmacist (in a neighborhood with the highest medical concentration in Paris) and the Carvallo brothers' father was a graduate of the Ecole Polytechnique and was a civil engineer. This elitism was matched by a strong cosmopolitan slant. Religious minorities (Protestants and Jews) were greatly overrepresented as were foreign pupils. Within this group, the largest body were Polish but Pierre Albertini notes the presence of Romanians, Germans, Austro-Hungarians, Swiss, Portuguese, a few Ottomans, British (or Franco-British), Americans and many South Americans. Among the names mentioned by Georges Bertrand, there is no surprise in seeing Jewish names (Carvallo), American ones (the Grant brothers) and British (Swann)^13^.

These characteristics were not without effect on the atmosphere that reigned at Condorcet. A teacher from Eton, on a visit in 1865, noted that it was “the most fashionable lycée in Paris” (Albertini, 2017) where social differences were particularly pronounced, liberalism was encouraged and snobbery highly popular. Taking this environment into account it is easier to understand the attraction which pastimes from across the Channel held for 1,879 schoolboys probably on the lookout for leisure time novelties, and the latest fashion from Brighton did not leave them unmoved. Alfred Swann was probably seen by his pals as being at the cutting edge of modernity because of his ability to satisfy their Anglomania.

The second original feature of the Lycée Condorcet lies in the fact it was only a day-school. Unlike all the other Paris state-run lycées, which subjected their pupils to the strictest of disciplines, with no outings possible, Condorcet provided a more flexible atmosphere and also the young people were able to leave the lycée. This relative freedom was the absolute prerequisite for meeting of the Société des Courses to be held in the nearby Bois de Boulogne. The boys who belonged to the club saw each other in the evenings after class and also on Thursdays and Sundays, and enjoyed free time which they could use for organizing athletic games.

### Youthfulness and the Dissemination of Sports

The group of young people who discovered football and athletics alongside Alfred Swann was a gathering of adolescents. In 1879–1880, Alfred Swann was 16–17, the same age as his schoolmate Oscar Carvallo, while this friend's brother Julien was only 13. No longer children and not yet young adults these lycée pupils match perfectly the definition of the nineteenth century adolescent according to Agnès Thiercé's research on the topic. This age is an element to be taken into account in the analysis of this phenomenon of the spread of sports. Within a French population which was ignorant of athletic sports coming from the British Isles the fact that it was young people who were behind such wide-ranging acculturation is not insignificant. Research into immigration and integration of populations into a new cultural context does in fact show that the younger the individual, the easier his or her acculturation in the host country. It might be surmised, beyond the fact that while sports require physical activity which better suits younger people, that they also have a certain open-mindedness linked to the socialization process which is still going on and which makes them more likely to catch on to new practices.

Research carried out in youth sociology can also be called on in order to understand the horizontal transmission of sporting practices between Alfred Swann and his school chums. In fact, these youngsters organized themselves, entirely independently, having chosen one of their number to be the initiator. This tradition of free organization and pupils being responsible for themselves is at the heart of the teaching ethos in British public schools. But above all this is possible because of their age: most of the members of the Société des Courses du Bois de Boulogne were aged 16–17 so it may be considered that they were able to achieve what Berger and Luckmann call secondary socialization, which comes after the primary stage and allows individuals to acquire skills and expertise through peer to peer relations and not just from their parents or other institutions incarnated by adults (school, church…) (Berger and Luckmann, 2006). This first acculturation through sports for and by the young is also to be found in other groups of sports participants who met in the Bois de Boulogne at the end of the 1870's and in the early 1880's, in particular the Racing Club de France (Bourdon, 1906).

Youth and the age criterion therefore need to be taken more into account in the analysis of sporting cultural transfers.

### Camaraderie or Manly Friendships?

The last element to be examined regarding this topic of the members of the Société des Courses du Bois de Boulogne concerns the friendly relations between the young men which were likely to foster the spread of sports. It is not possible to imagine the weekly encounters between the lycée pupils in the same meeting-place and the exchanges that took place without taking into account the degree of closeness that existed between them.

Affinities between schoolboys were the object of ambiguous views and judgements in the nineteenth century. At a time when there was strict segregation of the two sexes, “particular friendships” were feared and this Specter haunted teachers and school heads (Thiercé, 1999). But at the same time pedagogy was still partly shaped by Rousseau, whose influence was significant, and it set value on closeness: according to Emile “le premier sentiment dont un jeune homme élevé soigneusement est susceptible n'est pas l'amour, c'est l'amitié” (“the first sentiment of which the well-trained youth is capable is not love but friendship”) (Rousseau, 1911, p. 181). In the same way, many people saw in friendly relations a powerful means of flowering development and socialization. In any case, this is what seems to have happened in the case of the Société des Courses du Bois de Boulogne where the young knew and frequented one another and where they shared an activity.

The question therefore is whether one should speak of their friendship or camaraderie, the two words being frequently employed at the time with not much distinction between them, but they refer to real situations which were not exactly the same. Friendship implies a certain degree of affection (which goes beyond empathy) and a relationship which is very often dual in nature. Moreover, as Agnès Thiercé points out, in the nineteenth century “friendships in lycées were very strong. They were often close and long-lasting because they were rooted in a shared life” (Thiercé, 1999, p. 189). These dimensions, both affective and temporal, seemed to have an effect on some of the members of the sports club. In his article Georges Bertrand stresses that 20 years on he had not lost touch with three of his playing partners. As for Alfred Swann, he had a very deep friendship with one of the first members of the Racing Club de France, Georges Bourdon, who was the witness at his marriage in 1903 and the two men used to move in the same literary circles^14^. Thus, friendship can be one of the not unimportant sources for the spread of sports as Pierre de Coubertin recalled when he regretted the mistrust with which it was viewed, thinking as he did that, on the contrary, friendship can be one of the most powerful channels of education (de Coubertin, 1889, p. 10).

However, it seems to us that the idea of camaraderie better characterizes the relations within the group of lycée pupils. Firstly, because many of the members lost contact with one another thereafter or could not remember the names of their teammates and such forgetfulness is difficult to imagine in the case of friendship. Also, the very definition of the word seems to us to be better suited to the relationships that these schoolboys had, as a school friend is “a companion with whom one shares a common activity (studies, leisure pursuits)^15^.” Camaraderie does not require a strong and emotional closeness but implies acquaintance with the other and above all a shared activity, which here was sport. If we add the fact that we often speak of a playmate or a schoolfriend, we can understand that Georges Bertrand recalls his discovery of football in the company of “former sports mates” (La Presse, November 18, 1902). A final element which could be deployed in favor of camaraderie is manliness. In fact in the nineteenth century the comrade was first to be found among members of the same sex and the relationship with him helped to forge the basic qualities of a man^16^. So, inherent in the practice of athletic sports is the encouragement of the manly qualities and relationships to be found in clashes, courage, strength and stamina. Here in the past we find traces of the types of lycée cultures studied by youth sociologists. Dominique Pasquier suggests that friendly relations between girls “are based more on pairs or very small groups (the famous ‘Best Friend’ scenario) and they function along the lines of sharing intimacy” while masculine sociability at lycée “is based around groups and on the principle of shared activities [.]. The collective practice of activities such as sports or video games means that the principles of competition and hierarchy which characterize masculine groups are reaffirmed” (Pasquier, 2005, p. 279).

## An Acceptable Form of Sociability for Young People

### The Pioneering Société Des Courses Du Bois De Boulogne

The fact that lycée boys aged 13–17 were meeting up with no adult present and with the aim off doing physical exercises introduced from abroad and which were reportedly violent does not seem to have met with any opposition on the part of their parents. This seeming lack of any reaction cannot be put down to any kind of negligence involved in the education of these adolescents seeing how much importance the ruling classes paid to their offspring's' education, to their acquaintances and to their eventual careers. The legal guardians of the lycée pupils were anyway perfectly aware of these sporting practices which used to take place on the Pelouse de Madrid grass in the middle of the Bois de Boulogne. Since this wood had been transformed into a veritable Parisian Hyde Park during the Second Empire, it had become “the epicenter of Parisian high society” (Hopkins, 2003, 2015), whose denizens were in the habit of going there on a daily basis to take a stroll around the lakes. The gathering of young sportsmen around Alfred Swann thus took place in full view and with the full knowledge of their parents or at least of some of their acquaintances.

This acceptance of a form of independent and sporting adolescent sociability seems to have been most original in a context of distrust regarding gatherings of young folk. Adolescence, as a time of life set between childhood and adulthood, in fact provoked a real distrust among French elites in the nineteenth century (Thiercé, 1999). It was associated with the notion of crisis, the crisis of sexuality, linked to puberty, but also the crisis in morality as the adolescent was considered as being progressively inhabited by an enthusiasm, excitement and fervor which made him swift to rebel and to being ill at ease, or even prone to sexual deviancy. French educators and doctors in the first two thirds of the nineteenth century thus insisted on the need for strict control of the young. It was a matter of occupying, supervising and isolating individuals and this role fell chiefly on the educational institutions: the very many hours of lessons and the permanent supervision of the young, in particular in the case of boarders, was part of this strategy of warding off adolescent vices. Wariness of pupils also concerned their meetings: pupils' gatherings was widely seen as being despotic, vice-ridden and vulgar and helped to disseminate “a bad spirit” among group members this being characterized by war against authority and potentially shameful pleasures. In this regard, the years 1870–1880 constituted the apex and the beginning of the decline of this vision of adolescence. Agnès Thiercé in fact shows how a pedagogical turning point took place giving way to a more liberal attitude at the end of the 1880's and the beginning of the 1890's. More attention was being paid to the conditions in which the adolescent personality might flourish and a partial opening of lycées onto the outside world was begun. Young people's clubs and especially sports societies were also valued as they allowed pupils to be responsible for themselves and they acted as a propedeutic for their future role as citizens.

However, the Société des Courses du Bois de Boulogne appeared 10 years before these changes; the years when Alfred Swann and his pals got together and exercised freely in the Bois de Boulogne were also the years when many violent lycée revolts took place in Paris. Agnès Thiercé counted over 150 of them in the second half of the nineteenth century with a peak in the 1870–80 decade. How else then can the early establishment of this sports club and the freedom that its members enjoyed be explained?

### Anglophilia at the Lycée Condorcet

Over and above the special material conditions which the pupils at Condorcet enjoyed, in particular the fact that it was a day-school, one of the major reasons which allowed the existence of the early sports club, was due to the Anglophilia that pervaded this institution. In the conclusion of his study of the lycée in 1867, Pierre Albertini noted that the main characteristics of the lycée (liberalism, elitism, snobbery, and cosmopolitanism) were all so many components of a kind of Anglomania. The historian noted this about Condorcet: “They love almost everything about England, the fact that she has known how to preserve her monarchy and her aristocracy while exploring the paths of economic modernity, her model of parliamentarianism, her religious tolerance, her reformism, her openness to the world, the unconditional political asylum she offers to the outcast, her liking for sport, the gentlemanly conduct of her elites, and even Gladstone and Disraeli, who began their alternance in that same year of 1867” (Albertini, 2017, p. 89). There was therefore a close link between the Anglophile atmosphere which reigned in the neighborhoods of western Paris at that period, the spirit of the Lycée Condorcet and the sports played by the young men around Alfred Swann.

In the educational domain, this British influence led to the pupils being granted greater liberty (day-school, free association tolerated outside the establishment) and value being placed on physical activities. Interest in the school system across the Channel and the British model in general did not in fact date from the 1880's even if the successful novels by Paschal Grousset (alias Philippe Daryl) and the “pedagogical crusade” brilliantly headed by Pierre de Coubertin led to decisive steps being taken. In fact one has to go back to the end of the Second Empire to find traces of research into schooling on the other side of the English Channel and the sporting activities that were so prized. They were developed by doctors and educators seeking for a better balance between intellectual and physical activities as well as teaching that would form entrepreneurial elites. As early as 1867, Victor de Laprade, author and teacher of literature, published *L'Education Homicide*, a famous pamphlet in which he savagely criticized lycéens' living conditions, where they remained seated listening to their teachers for 11 hours a day (de Laprade, 1868). Basing his comments on the British example, he proposed reducing this teaching by half and using the time gained for the pupils to get fresh air and play (de Laprade, 1868, p. 114–115). These recommendations were published the same year as a report by two lycée teachers on their return from a pedagogical mission to Britain (Demogeot and Montucci, 1867). The authors write of the essential role played by sports in the public schools and insisted on the “considerable” advantages of these activities which reinforce energy, courage and the desire for glory (“here we see at its source the flow of a true and legitimate aristocracy” (Demogeot and Montucci, 1867, p. 22), even though they judged that the time spent on open-air sports to be excessive and thus harmful to intellectual performance. Their conclusion invited the decision makers at the Ministry to reflect on the means required to integrate more physical activity into lycée life without falling into the excesses seen across the Channel.

The influence of these early accounts and reports of the British educational model did not at first go beyond the limited circles of those interested but the events of 1870–71 gave them an unexpected boost. The terrible French defeat by the Prussians, which brought about the fall of the Second Empire and was a national humiliation, in fact stimulated a number of discourses and reflections seeking to build France, ever stronger and able to prepare for Revenge on Germany, while more generally re-establishing a global ambition of the first order. Among the vital forces to be regenerated, the young were a priority and in particular the elites of the Republic, formed in the lycées. Educators and essay writers were therefore interested in the education systems of neighbors which might provide inspiration and in particular in the cases of Germany and Great Britain (Matasci, 2015). While many pieces appeared in the 1880's, two major essays were published in the preceding decade: *La Réforme de l'Enseignement Secondaire (Reform of Secondary School Teaching)* by Jules Simon (Simon, 1874), professor of philosophy and government minister but especially *Notes sur l'Angleterre* by the historian and essayist Hippolyte Taine in 1872 (Taine, 1872). He was a liberal like Jules Simon, and had authored a particularly influential piece. This was due initially to his personality and to the context, for at the beginning of the IIIrd Republic Hippolyte Taine was a famous intellectual who wished to study and understand the *Origines de la France Contemporaine*, but who also sought, in British Otherness, the keys for analyzing and reforming his country. His *Notes sur l'Angleterre*, written just after the 1870 defeat came therefore from a renowned intellectual and met with instant success with the public. This success was all the stronger at Condorcet where Taine enjoyed a most flattering reputation. He had been a pupil there and his school career had been remarkable. With Henri Bergson and Théodore Reinach, he was one of the great French intellectuals educated at Condorcet and his influence in that establishment was particularly strong. Yet, what does he tells us in Chapter IV of his *Notes sur l'Angleterre* entitled “Education?” That he admires the house system that operated in British public schools for it was not reduced to “a barracks communism like in France” (Taine, 1872, p. 136); that the fresh air which the pupils at Eton and Harrow enjoyed was in contrast with the lack of greenery and the confined spaces of French lycées; that the pupils' freedom gave them a sense of initiative and responsibility; that the sports played were the logical response to a “need for physical movement” felt by the pupils and this helped them to learn how to command as well as how to obey. His conclusions are however more subtle because while he recognized the ability of British establishments to forge the character of their pupils, he regretted the weakness of theoretical teaching and the “basic instincts” (Taine, 1872, p. 143) that an excess of sport awakened in the boys. In short, a fusion of French intellectual excellence and the apprenticeship of life so dear to British public schools would lead to a school system that was well-balanced and of quality.

### New Educational Methods and the Spread of Sports

The situation regarding the members of the Société des Courses du Bois de Boulogne seems very much like a practical application of Taine's recommendations. Attending a French lycée whose academic reputation was impeccable, their twice daily sorties and their frequenting the Bois de Boulogne under the auspices of a free and autonomous association seemed to incarnate to perfection the school-life balance preached by the glorious former pupil. Moreover, since 1875, they also had access to the French translation of Thomas Hughes's famous novel, *Tom Brown's Schooldays*, which Hippolyte Taine highly recommended (Hugues, 1875). There is no doubt that the parents of many pupils at Condorcet were already won over to the liberal cause and to the Anglophilia of the Parisian elites and they doubtless looked with a kindly eye on their offspring affirming their character and liberating their adolescent energies yet without jeopardizing their scholarly success.

Outside Condorcet, the first groups of pupils playing sports in the Bois de Boulogne at the end of the 1870's systematically belonged to establishments offering innovative teaching methods, partly inspired by the British model. This was the case of the Ecole Monge, founded in 1869 and which had a Football Club from 1878, and also the Ecole Alsacienne, which opened in 1874 and where some of the pupils founded the Société Sans Nom (No Name Club) in 1880. The school and family environment in which these first players of British sports grew up played an important part in their acculturation insofar as it accepted and often encouraged the wish to take up these manly pursuits from across the Channel.

## The Inconvenience of Being Older: The Example of Cosme de Satgé

The discovery of British sports by an older person stands in counterpoint to the study of Alfred Swann and his schoolfellows and allows the emphasis to be more strongly placed on the importance of youthfulness and friendship or camaraderie in acculturation thanks to sports.

### Cosme De Satgé: A “Dual Figure” Within the “English Colony” in Dinan

Cosme de Satgé (1840–1898) was a Franco- British man of the second half of the nineteenth century whose singular story has recently been brought forward by the historian Diane Monier-Moore in her research into the English colony in Dinan (Monier-Moore, 2017). Cosme was born in Pau, the son of a French aristocrat, Antoine de Satgé (1807–1870) and a British woman of Irish origin, Harriet Rowley (1808–1892). He spent the early part of his childhood in the Béarn region and then in Ariège before being sent to Paris as a boarder at the Collège Sainte-Barbe (1852–58). He then studied law in Paris and obtained his doctorate in 1865. However, he never followed a legal profession and led the life of an idle aristocrat living off unearned income until the mediocre state of his investments pushed him into teaching French to British boys who boarded with him.

In 1872 Cosme de Satgé married Frances Knippe, a British woman from Stratford upon Avon, and they set up home in the small Breton town of Dinan, located about 30 km from the Channel coast. He lived there for 25 years, raising five children (Henriette, Yvonne, Philippe, Béatrice, and René, born between 1874 and 1885) before dying suddenly at the beginning of 1898. The Satgés' choice of Dinan in 1873 can be partly explained by its proximity to the British Isles but also by the presence of a large “English colony.” Drawn by the climate and the presence of restorative and therapeutic mineral springs, many British citizens had settled in this town in the Côtes d'Armor department during the nineteenth century. Out of the 8,000 or so residents in Dinan in the 1850–1860's about 400 were British (Monier-Moore, 2017, p. 36). This figure doubled during the summer season because of the number of holidaymakers who crossed the Channel. The colony consisted mainly of retired officers from the Indian Army or the Royal Navy, of families from the Caribbean who found themselves in difficulties because of the abolition of slavery or British widows and their children. All were eager to enjoy the pleasant setting of the little town of Dinan where it was possible to have a comfortable lifestyle at less cost than in Britain. The community, which mainly lived off investment income and pensions, rapidly organized themselves: an Anglican church, a Masonic lodge, an English library and a British club founded in 1868, were all progressively established and became pillars of a contained social life, living in the English style but on foreign soil. Playing games and sports also brought these expatriates together; cricket had already been established in the 1850's and lawn tennis was then introduced. Already in 1875 the colony newspaper, *The Dinan Weekly*, spoke of it being played on Tuesdays and Fridays “on the most seedy grass” (The Dinan Weekly, August 17, 1875). As for ice-skating, the nearby river Rance froze over for part of the winter providing yet another leisure activity appreciated by the British.

We know about the life that Cosme de Satgé led in Dinan thanks to an exceptional document: his private diary, most of which was written following his arrival in the Côtes d'Armor. It comprises 22 volumes, containing nearly 5,000 pages and is a remarkable first-hand account which helps to understand the way this “English colony” functioned in France in the nineteenth century while offering insight into the itinerary of a Frenchman in this British environment. While his mother was Irish and his wife English, Cosme de Satgé was basically a man of French culture. Besides his nationality his own story was situated in France where he was born, educated and where he had friends, before settling in Dinan. His visits across the Channel, though frequent, did not begin until he was 19 and his lack of knowledge about athletic sports testifies to an incomplete Britishness. However, moving to Dinan with his mother and his wife plunged him into the world of the “English colony” though it was not exactly open to local people. The British people in the town in fact mainly existed in a vacuum all the more so as none had any local roots. Most had arrived in France at some point in their lifetime and did not work there, living off unearned income and pensions. Those who did have a profession carried it on within the community, like the dentist, the doctor, the grocer and even the Anglican clergyman. As for the few children present, most went to private schools in the colony like Ker Even and were then sent back across the Channel to continue their secondary education. Thus, there was no strong desire for integration as can be borne out by the very few mixed marriages celebrated in the Anglican chapel during the nineteenth century, as well as the colony members' poor command of the French language. Relations between French and British were not non-existent though because day to day life involved meetings and relations with local administrative, political and religious authorities. This is why there was a point in the colony's having a go-between who was both bilingual and well-versed in the habits and customs of the Hexagon, France. Cosme de Satgé was progressively called upon to bridge the gap. Thanks to his perfect command of English but also and above all thanks to his wife and mother he wove a relatively dense network of relationships within this colony which took him into its inner circles and in return it was requested that he help with its functioning. His services were called on when a delegation went to see the député-maire (mayor and member of parliament) of Dinan to get permission to open an English school, to protest to the sub-prefect when the British Club was accused of having organized clandestine gaming, or more prosaically when it came to hiring a servant or finding a lavatory for the circle (de Satgé, 21 January 1880, 30 July 1883, 29 November 1884, 9 February 1886, 9 November 1886). His affability as well as his knowledge of national legislation and the workings of French administration—which already irritated the British to quite some degree—encouraged many families to ask his advice.

### Playing and Passing on Youthful Pursuits

Among the sporting leisure pursuits in which Cosme de Satgé indulged, a distinction should be made between those he had discovered during his youth and those he found at Dinan when he was an adult. He regularly participated in the former and these pastimes were passed on in the family. Such is the case for horse-racing which is first mentioned in his diary in 1861. At that time Cosme de Satgé was 21 years old and studying law in Paris. He relates how he used to stroll through the Bois de Boulogne after class in summer and was not averse, from time to time, to going further, as far as the racecourse at Longchamp in order to see a few races with some of his friends. His later sojourns in the capital see him return to the western end of the Bois in order to bet on one or two top runners after having observed them during the weighing-in. Similarly, during his trips to Britain he would regularly visit British racecourses such as Southampton (in the summer of 1869) or Ascot (1870). As soon as he moved to Dinan in 1873 he became a member of the Société des Courses (Racing Society) (de Satgé year 1873). Although he was not as passionate about the turf as he was about hunting or skating, Cosme de Satgé regularly paid his fees to the local society and took his children to the local racetrack at Aublette on several occasions.

Ice-skating on the other hand was a true passion for our Franco-Englishman. His diary has several entries relating to the wait for the first freezes and the hope of a hard winter, and neither age nor weight curbed his enthusiasm. It must be pointed out that he had cherished this pastime since his years at the Collège de Saint-Barbe where the harsh regime of the boarding school left him few free afternoons when he could go to “the Bois” with his pals. Thus, it was on the ponds of the Parisian Hyde Park that he took his first skating steps and the pleasure he found in this would never leave him. In December 1873, freshly arrived in Dinan he took advantage of a trip to the capital to go to the Cercle des Patineurs (Skaters' Circle). There, he decided to buy a pair of skates in the hope of being able to practice in Dinan (de Satgé, 13 December 1873). While it was not possible for him to skate on the river Rance during the first Breton winters, the month of January 1897 was particularly cold. Cosme de Satgé copied the British in the town and discovered the best spots for skating. He practiced using the edges of his blades and discovered ice-hockey during a game with “English officers” (de Satgé, 9 December 1879). He cherished the idea of sharing this activity with his children. As early as January 1889 he began to teach his two eldest children, Henriette and Béatrice, and he did not take long to buy them skates. The two girls were then aged 15 and 13. The following winter it was Philippe on the eve of his 11th birthday who discovered the joys of winter sports alongside his father. In his journal Cosme de Satgé recounts the wait for the cold weather, then his impatience while waiting to fetch the children from school to take them to the Rance, despite the fact that he was over 50 years old. However, the pastime that Cosme de Satgé enjoyed the most and the one to which he devoted the most time was hunting; not hunting with hounds (the famous British fox hunting) but carried out on foot, which gave rise to long walks which could take up the whole day and where the trophies bagged (hares or birds) were almost secondary in his eyes because for him pleasure was being in the open air. His earliest hunting memories came from when he was in his 9th year and his father took him for the first time out on this manly pastime which could be solitary or else a shared experience (de Satgé, volume 1). This transmission, vertically from his father contrasts with his acculturation thanks to skating but it marked him all the same. He would also recall clearly gaining his first hunting permit in 1856 (at the age of 16) and of hunting parties in France as well as in Ireland and England. Settling in Dinan offered him the chance to indulge in this pastime which he did between once and three times a week during the season, which is in autumn and winter. Sometimes he went out hunting alone but he liked company and that of the British in particular. Major Hinchcliffe, whom he met for the first time in February 1875 and who accompanied him every week thereafter over several seasons, became a real friend of Cosme de Satgé (de Satgé, 1 February 1875). The hours they spent together walking and talking, between two shots were the occasions for sharing memories, opinions and close chats. Cosme de Satgé actually only used the letter H to refer to his companion whereas otherwise the rare abbreviations in his journal were reserved for his wife and his mother. Afterwards, Hinchcliffe became the godfather to his second son and when the major left Dinan this deeply saddened the Frenchman who nonetheless kept up his activities and began to initiate his son Philippe, who initially followed his father along the path on a tricycle and eventually went all the way on foot. These were special moments shared between the father and the young man who seemed to take a liking for hunting, since according to the last years of Cosme de Satgé's diaries increasingly Philippe was going out game hunting alone.

The common point between these three leisure activities, horse-racing, ice-skating, and hunting, was that Cosme de Satgé discovered them early in life, through a precocious acculturation which manifested itself through regular practice and being passed down through the family. However, the arrival of the Franco-Englishman in Dinan and his acquaintanceship with the “English colony” was the opportunity for him to discover pastimes introduced from over the English Channel.

### The Discovery of New British Sports in Dinan

Like many other intimate writings, Cosme de Satgé's diary remains a personal document, above all factual, in which he notes down the activities and high points of his day. In the tone of the accounts set down in these journals and the few impressions that the author lets through and the very nature of his timetable, it is possible to outline the elements of his personality. Three character traits in particular arise from reading these notes: his relative humility, his graciousness and his simplicity.

Although he was nobly born and above all idle, he did in fact try to lead a peaceful life, as is evidenced by his reading sessions either alone or out loud to his children, and his taste for gardening. Moreover, there was nothing of rebel vanity about him; the tiresome administrative tasks that he took upon himself to aid the English colony and the many visits paid or received at teatime or for a picnic bear witness to this civilized character who voluntarily submitted to the demands made by his entourage. This made him a man who was relatively open to new practices and not much inclined to wholesale, swift rejection, as is borne out by his relation to cricket and golf. Even though he does not mention it in his journal it is most likely that he knew these two sports before 1870, the date at which he notes that he had attended a cricket match during his first visit to Dinan. But even though his curiosity made him an interested spectator, he nevertheless never played these games. His daily strolls with his wife often took him close by the players of the United Dinan Cricket Club, practicing during the high season. This spectacle seems to have been part of the town décor for Cosme de Satgé. Several times he mentions games that were going on but he obviously did not know the rules and was unconcerned about the result. The same went for golf which was played on the Breton seashore and which he happened to watch during his summer holidays in July or August in Dinard or Saint Lunaire. In July 1891, relating a stroll with his daughter he noted this: “With Henriette, followed a game of golf on the dunes near the Hotel du Panorama. The game interests us. Interrupted by light drizzle” (de Satgé, 26 August 1891). Never having encountered these sports other than as a spectator the attraction for him never went beyond simple, occasional curiosity all the more so since these games are marked by long pauses which are not likely to incite sustained interest. Beyond the swift mention of these sports when he was out on a walk, it was Cosme de Satgé's involvement in the life of the English colony or his local friendships which brought him onto the golf course and cricket pitch. So, his election to the British Club in 1879 came with obligatory membership of the Cricket Club. That same year, in August, he attended a match, noting “Everyone we knew was at the cricket match—interesting game—Dinan seem to have beaten Avranches” (de Satgé, 6 August 1879). Similarly, when he accompanied his friend Colonel Garnett to Dinard in August 1894 he followed him to the Golf Club where he was present at “the beginning of a game” along with many leading members of the local English colony (de Satgé, 28 August 1894). There was therefore no rejection of these leisure activities but his relation to golf and cricket remained platonic.

This was not the case for tennis which he discovered in the course of 1879. After having seen a member of his family play at a Lawn Tennis Club, in June during a stay in England, on 4 July he went to a garden party given by Major Young in Dinan. He writes “Game of lawn tennis. M Chevallier, a beginner, like me. Very nice game, but rather hard.” This first contact seems to have been quite conclusive because straight away Cosme de Satgé paid his membership fees at the Dinan Lawn Tennis Club and he played on the club courts on 26 July. He also took advantage of the presence in Dinan of Tottie, his British sister-in-law, in order to play against her in the garden. The first games were deemed difficult with “not much success” (de Satgé, 10 September 1879), but seven matches are noted in his diary for the second fortnight in August. The pace flagged somewhat in the autumn, the end of the fine days, allied with the departure of his sister-in-law and the obligation to leave his house in the Mont-Parnasse neighborhood for another rental in town, which left Cosme de Satgé without a regular tennis partner (his wife was pregnant and little inclined toward sports) and no garden to play in. This dual loss, both in relational and spatial terms seems to have been fatal for his acculturation. Certainly, he continued to play lawn tennis from time to time, such as in the autumn of 1882 or in August 1885 when he met up with his sister-in-law during trips to England (de Satgé, 6 October 1882 and 18 August 1885), but these events were only occasional and de Satgé seems to have played to please his partners. In this semi-failure the absence of a regular playing partner (someone like Major Hinchcliffe for the hunting) and his relatively late discovery of this sport (he was 39 in 1879) seem to have been the main causes. However, we can note that visits to Dinan by his few French friends were always the occasion for Cosme de Satgé to introduce them to the sporting curiosities of the English colony. When his former collège chum Chautard spent a few days at the villa at Mont-Parnasse in August 1873 he was taken “to see a cricket match^17^.” In the same way, Pettereau, a childhood friend was taken to the lawn tennis courts in 1885: “there was in fact a good match going on and the game interests him^18^.” Cosme de Satgé is therefore positioned as a cultural go-between in that he offers his friends a first visual contact with the sports along with a few introductory remarks. Moreover, he contributed to the successful acculturation of his children regarding British sports.

### The Successful Sporting Acculturation of the Satgé Children

Unlike their father, Cosme de Satgé's five children, who were born in Dinan and grew up within the English colony, enjoyed both an early acculturation and relatively many playmates. Their case is interesting insofar as their mother, although British, was not a sportswoman and their father was only able to pass on them his enjoyment of hunting or skating and his taste for horse racing. While he gave them a liberal education and was not above joining in their tennis matches, at best he can be considered as a facilitator and not as a mediator regarding British sports.

By what means then did these sports pass among the young people? In the first instance there were invitations from other families in the English Colony. Picnics brought together a number of guests and were occasions for playing together, like in July 1892 at the home of the Filgates where “the young ones played cricket” (de Satgé, 21 July 1892). But the invitations were also individual and Cosme's children played tennis several times at their friends' homes. Besides, all the de Satgé girls went to school at Ker Even, a British institution, where racket sports were played by pupils. The children's sporting culture was also part of their holidays across the Channel. In August 1885, Henriette, aged 11, played tennis with her aunt and her father in England and “begins to understand the game” (de Satgé, 19 August 1885). Her sisters then in turn went to visit their mother's family from 1890 onwards. This British influence on the children was further strengthened from 1893 when Cosme de Satgé decided to take as paying guests students from across the Channel who came for him to teach them French. The young people, who spent between 2 and 6 months with the family, got on very well with the de Satgé sons and daughters. Thus, in 1895, the Osbourne brothers who did not distinguish themselves by their application to learning the French language, encouraged Philippe and Yvonne to play tennis (de Satgé, 20 June 1895). There were many students who passed through Dinan who paid their membership fees at the Lawn Tennis Club. However, the tennis virus, and cricket for the boys, was really caught on the beaches of Brittany where the de Satgé family spent several weeks each summer. Cosme de Satgé and his wife nevertheless provided a range of activities for their children, from sea bathing to fishing, walking on the dunes and board games. Several summers passed without tennis or cricket playing a part in the daily life of the de Satgés. But, it is also true that the family spent time on holiday with British friends, in 1892 and 1893 with the Forrests, whom they met up with several times on the beach. Tennis and cricket matches are then frequently mentioned in Cosme de Satgé's diary. The turning point which saw the de Satgé children definitively adopt tennis and cricket was in the summer of 1896 on the beaches of Saint Lunaire in the company of the Bateson sons and daughters. The youngsters, busy with their rackets and bats, thus refused to go fishing with their father (de Satgé, 9 September 1896). This attitude can be put down to a kind of adolescent autonomy but also a successful acculturation through sports. The following year most of the de Satgé children became members of the Dinan Lawn Tennis Club. They were then aged between 12 and 23 and both boys and girls enjoyed this leisure activity.

## Conclusion

This study aimed at highlighting the need to combine general cultural factors with methodological individualism in order to better understand the ways in which British sports spread in nineteenth-century France. In this process, one must try to analyze the different variables that make the chain of dissemination possible. The dual identity of the cultural go-betweens is often a prerequisite without which no bridge can be built between two cultural areas. But it proves unsufficient to fully understand the motivations of these brokers and must therefore be completed by other elements. On the other hand, recipients are not as passive as one could think. They forge links and build relationships with their donors. To use Sébastien Darbon's expression, historians, anthropologists and sociologists interested in sports diffusion must take into account what is “in the way of being acceptable (or inacceptable), compatible (or incompatible)” among those individuals who are faced with a new cultural product (Darbon, 2008, p. 5). Some studies can emphasize more or less favorable national contexts but also get down to the level of the individual actor in order to understand what, in the story and the personality of some people, can explain the enthusiasm or the rejection of certain sports. If Anglomania (or Anglophobia), religious practice or even professions are often advanced, other criteria are sometimes underestimated.

This study of two Franco-British cultural mediators and their entourage has insisted on the importance of age and personal affinities in the process of acculturation through sports. Despite their dual culture Alfred Swann and Cosme de Satgé did not adopt British pastimes with the same enthusiasm: the former discovered modern sports when he was an adolescent and became an active agent in their spread in Paris whereas the latter did not manage to truly appreciate and pass on the British leisure activities he discovered after he had turned 30.

Moreover, the spread of sports often follows the channels of friendly relations and camaraderie. The young lycée pupils around Alfred Swann, like Cosme de Satgé's children, were acculturated through British sports at the time of their adolescence, not by adults, but by their friends and chums of their age: friendship and camaraderie favor the desire in some to share a pastime and the desire in others to open up to a new activity. The spread of sports is thus a condition as well as the culmination of personal relations between those who transmit and those who receive.

Underlying the importance of age and the role of interpersonal relationships in the process of sports diffusion opens new avenues of research for the future. To a certain extent, the relevance of these two factors could lead sports historians to reassess some analyses: youth, camaraderie (or friendship) and horizontal dissemination could offer both a new angle of approach, and a broader look at subjects such as cultural mediators and sports clubs.

## Data Availability Statement

The original contributions presented in the study are included in the article/supplementary materials, further inquiries can be directed to the corresponding author/s.

## Author Contributions

The author confirms being the sole contributor of this work and has approved it for publication.

## Conflict of Interest

The author declares that the research was conducted in the absence of any commercial or financial relationships that could be construed as a potential conflict of interest.

## Footnotes

^1^Proust (1913)

^2^De Satgé C., Private Diary, owned by the de Satgé family in London.

^3^In particular, see Bourdon Georges, La Renaissance athlétique et le racing Club de France, Paris, published by the RCF, 1906. Also, Daryl (1894). Finally, Blum (1924).

^4^Paris Archives, Civil Register, Register of births in the 1st arrondissement, year 1863, registration n°696.

^5^His father first lived in Rue des Belles Feuilles, then in Avenue du Bois de Boulogne.

^6^Probate Registry year 1899, page 121.

^7^*Bulletin de la Société du Livre d'Art*, Paris, January1904, page 2.

^8^*Le Guide du Concert*, n° 28 and 29 (25 April and 3 March 1911).

^9^Paris Archives, Civil Register, register of marriages in the 8th arrondissement, for the year 1885, registration n°364; for the year 1893 registration n°929 (divorce ruling).

^10^*Alphabetical list of the children of foreigners having turned down or given up French nationality*, Paris, Imprimerie Nationale, 1946, page 523.

^11^*Racing Club de France directories*, years 1885–1914.

^12^However it was not the only secondary school in the smart areas of Paris because there were also two municipal collèges (middle-schools) (Rollin in the 9th arrondissement and Chaptal in the 8th arrondissement), as well as a private school on Boulevard Malesherbes: l'École Monge.

^13^Paris Archives, folder D4T3 96 (admission registers of Lycée Condorcet, year 1878).

^14^Paris Archives, Civil Register, register of marriages in the 17th arrondissement, year 1903 number 112.

^15^*Le Petit Larousse Illustré*, 1994.

^16^Vincent-Buffault (1995)

^17^Vincent-Buffault (1995), 8 August 1873

^18^Vincent-Buffault (1995), 8 August 1885.
